# Comparative Insights into Digestion and Gut Microbiota Modulation of Polysaccharides from Ginseng, Ganoderma lucidum, and Dendrobium officinale

**DOI:** 10.3390/foods15111921

**Published:** 2026-05-29

**Authors:** Xiaohua Pan, Wenyao Zhang, Weiwei Wang, Zhonglei Wang, Fanglan Li, Chang Liu, Rongrong Ma, Yaoqi Tian

**Affiliations:** 1 State Key Laboratory of Food Science and Resources, Jiangnan University, Wuxi 214122, China; 2 School of Food Science and Technology, Jiangnan University, Wuxi 214122, China; 3 Instrumental Analysis and Laboratory Animal Center, Jiangnan University, Wuxi 214122, China

**Keywords:** polysaccharides, structure, digestion, gut microbiota, carbohydrate-active enzymes

## Abstract

Dietary polysaccharides regulate gut microbiota and exhibit diverse prebiotic activity, which is highly dependent on their structural properties. To explore the underlying structure-prebiotic relationship, this study selectively compared the structural characteristics of *Ginseng* polysaccharide (GP), *Ganoderma lucidum* polysaccharide (GLP), and *Dendrobium officinale* polysaccharide (DOP) and investigated their digestive stability and gut microbiota modulation via *in vitro* simulated digestion and fecal fermentation. Structural analysis revealed distinct differences in molecular weight, monosaccharide composition, and glycosidic linkages among the three polysaccharides. Moreover, GP is partially digested in the upper gastrointestinal tract, while GLP and DOP were resistant to upper-tract digestion. All three polysaccharides differentially modulate gut microbial fermentation, intestinal microbial community structure, and the expression of functional carbohydrate-active enzymes. Specifically, the high glucose content of GP selectively promoted the abundance of genera putatively linked to glucose utilization, including *Bacteroides*, *Bifidobacterium*, and *Alistipes*. GLP preferentially enriched possible genera with galactose-metabolizing ability, such as *Blautia*, *Collinsella*, and *Megamonas*, while DOP selectively enriched microbiota putatively associated with mannose utilization, including *Fusicatenibacter* and *Lachnospiraceae*. Taken together, monosaccharide composition is a key structural feature that is closely associated with fermentation efficiency and gut microbial responses to polysaccharides, providing valuable insights for the precision utilization of bioactive polysaccharides.

## 1. Introduction

Polysaccharides are complex carbohydrates composed of homogeneous and heterogeneous monosaccharides, which are connected by specific glycosidic linkages [1]. Recently, polysaccharides have attracted extensive attention owing to their favorable biocompatibility, low toxicity, and multiple biological functions, such as intestinal barrier protection, immunomodulation, and metabolic regulation [2]. Emerging evidence suggests that most bioactive polysaccharides resist direct digestion and absorption in the human upper digestive tract and are mainly decomposed by the bacteria in the large intestine, as the mammalian genome lacks the enzymes required for such processes [3,4] and gut microbiota exhibit an array of carbohydrate-active enzymes (CAZymes) [5]. In this context, the gut microbiota act as a central mediator linking dietary polysaccharides to host physiological homeostasis.

Functional polysaccharides alter the microbial composition differently, owing to their diverse structures and specific microbial CAZyme profiles [6]. Structural properties, such as monosaccharide composition, molecular weight distribution, glycosidic bond linkage and tertiary conformation [7], are recognized as key factors affecting the enrichment of specific microbiota and the physiological effects of various polysaccharides [6]. *Ginseng* polysaccharide (GP), *Ganoderma lucidum* polysaccharide (GLP), and *Dendrobium officinale* polysaccharide (DOP) are three representative polysaccharides isolated from fungi or plants, with well-known prebiotic and physiological activities [8]. Existing studies have separately revealed their antioxidant, anti-inflammatory, and microbiota-regulating capacities. However, a systematic comparison of their structural differences and the intrinsic correlation between structure and microbial utilization of GP, GLP, and DOP remains insufficient.

To explore the structure-digestion-microbiota regulation relationship of three typical natural polysaccharides, the present study characterized the structural properties of GP, GLP, and DOP; compared their dynamic changes throughout simulated oral, gastric, and intestinal phases of digestion; and investigated their regulation of fermentation performance, microbial community, and functional CAZyme profiles. Our findings provide theoretical support for the implementation of GP, GLP, and DOP as functional prebiotic ingredients for gut health improvement.

## 2. Materials and Methods

### 2.1. Materials and Reagents

*Ginseng* polysaccharides (GP, #SR8580, purity ≥ 80%) were obtained from Solarbio Science & Technology Co., Ltd. (Beijing, China). *Ganoderma lucidum* polysaccharides (GLP, HY-159059, purity ≥ 75%) were supplied by MCE, Shanghai, China. *Dendrobium officinale* polysaccharides (DOP, #D708299, purity ≥ 80%) were purchased from Macklin Chemical Co., Ltd. (Shanghai, China). Pepsin (3000 U/g) and pancreatin (4000 U/g) were purchased from Sangon Biotech Co., Ltd. (Shanghai, China). Monosaccharide standards (fucose, galactosamine, rhamnose, arabinose, glucosamine, galactose, glucose, xylose) were purchased from Sigma-Aldrich (St. Louis, MO, USA).

### 2.2. Molecular Weight (Mw) Determination

To determine the molecular weight (Mw) of GP, GLP, DOP and their fermented samples, high-performance size-exclusion chromatography (HPSEC) coupled with multi-angle laser light scattering (MALLS; DAWN HELEOS 8+, Wyatt Technology Co., Santa Barbara, CA, USA) was employed. The chromatographic separation was carried out at 25 °C using 0.1 M sodium nitrate as the mobile phase, which was eluted at a flow rate of 0.5 mL/min. Prior to injection, GP, GLP, and DOP were dissolved in sodium nitrate solution at a concentration of 5 mg/mL and then filtered through a 0.22 μm membrane.

### 2.3. Fourier Transform Infrared Spectroscopy (FT-IR) Analysis of Polysaccharides

The GP, GLP and DOP samples were mixed with potassium bromide powder at a ratio of 1:100, respectively, pressed into tablets, and subjected to FT-IR analysis. The FT-IR spectra of polysaccharides were recorded using a Nicolet IS10 FTIR spectrometer (Thermo Electron Corp., Waltham, MA, USA) at a resolution of 4 cm^−1^ and a wavelength range of 400–4000 cm^−1^.

### 2.4. Determination of Physicochemical Properties of GP, GLP and DOP

The concentration of reducing sugars in the digestive and fermentation samples was determined using the DNS (3,5-dinitrosalicylic acid) method [9], and absorbance was detected at 550 nm with glucose as the reference. Total sugar content during *in vitro* digestion and fermentation was determined by the phenol-sulfuric acid method [10]. The pH value of fermentation products was recorded, and optical density at 600 nm (OD_600_) was measured using a microplate reader.

### 2.5. Monosaccharide Composition Analysis

The monosaccharide compositions of GP, GLP, and DOP were determined using high-performance anion-exchange chromatography coupled with pulsed amperometric detection (HPAEC-PAD; Dionex ICS-5000+ SP-5, Thermo Scientific™, Waltham, MA, USA) according to a previously described method [4]. Briefly, a mixed standard stock solution (100 mg/L) was prepared by dissolving monosaccharide standards in ultrapure water, including fucose, galactosamine, rhamnose, arabinose, glucosamine, galactose, glucose, xylose, mannose, fructose, galacturonic acid, and glucuronic acid. The stock solution was then serially diluted to obtain a series of mixed standard working solutions at concentrations of 6.25, 12.5, 25, 50, and 100 mg/L for calibration curve construction.

For sample preparation, 2 mg of each polysaccharide was dissolved in 2 mL of 4 M trifluoroacetic acid (TFA) and hydrolyzed at 100 °C for 5 h. After complete hydrolysis, residual TFA was thoroughly removed by nitrogen blowing. The resulting hydrolysate was redissolved in 2 mL of ultrapure water and filtered through a 0.22 μm microporous membrane prior to injection. Subsequently, 20 μL of the pretreated sample solution was analyzed by HPAEC-PAD. Chromatographic separation was achieved using gradient elution with 0.1 M NaOH and 0.2 M CH_3_COONa as the mobile phases at a flow rate of 0.5 mL/min.

### 2.6. In Vitro Gastrointestinal Digestion

The GP, GLP and DOP (5 mg/mL) were digested in simulated gastrointestinal digestion model following the INFOGEST protocol with slight modifications [11]. Polysaccharides were firstly mixed with an equal volume of simulated saliva (pH 7.0), which consisted of 15.1 mmol/L KCl, 3.7 mmol/L KH_2_PO_4_, 13.6 mmol/L NaHCO_3_, 0.15 mmol/L MgCl_2_·6H_2_O, 0.06 mmol/L (NH_4_)_2_CO_3_, 1.1 mmol/L HCl, and 1.5 mmol/L CaCl_2_·2H_2_O. The mixture was digested at 37 °C for 0 min and 5 min; samples were collected and rapidly heated to 100 °C for 5 min to terminate the reaction.

After simulating saliva digestion, the digested solution was mixed with an equal volume of simulated gastric juice (6.9 mmol/L KCl, 0.9 mmol/L KH_2_PO_4_, 25 mmol/L NaHCO_3_, 47.2 mmol/L NaCl, 0.12 mmol/L MgCl_2_·6H_2_O, 0.5 mmol/L (NH_4_)_2_CO_3_, 15.6 mmol/L HCl, 0.15 mmol/L CaCl_2_·2H_2_O, 2000 U/mL pepsin and 60 U/mL gastric lipase) and adjusted the pH to 3. The mixture was digested at 37 °C for 1 h, and samples were collected for further Mw, total and reducing carbohydrate analysis.

For intestinal digestion, the post-gastric residues were combined with an equal volume of simulated intestinal juice (6.8 mmol/L KCl, 0.8 mmol/L KH_2_PO_4_, 85 mmol/L NaHCO_3_, 38.4 mmol/L NaCl, 0.33 mmol/L MgCl_2_·6H_2_O, 8.4 mmol/L HCl, 0.6 mmol/L CaCl_2_·2H_2_O, 10 mmol/L bile salt, and 100 U/mL pancreatin), and the mixture was adjusted to pH 7.0. The mixture was digested at 37 °C for 2 and 4 h; samples were collected and heated at 100 °C for 5 min for further analysis.

### 2.7. In Vitro Fecal Fermentation

The *in vitro* fecal fermentation of GP, GLP and DOP was conducted following our previously reported procedure [12], which was approved by the Medical Ethics Committee of Jiangnan University (approval number: JNU202509RB013). Briefly, fresh fecal samples were collected from five healthy volunteers (three females and two males, aged 20–30 years) with no history of gastrointestinal disease, no antibiotic use in the previous three months, and no alcohol or probiotic supplementation for at least one month before sampling.

In an anaerobic chamber, the stool feces (2 g) from each donor were pooled in equal weight portions and thoroughly suspended in phosphate-buffered saline (PBS) at a ratio of 1:3 (*w*/*v*). This equal-weight pooling strategy was adopted to reduce the impact of inter-individual variability. Following thorough homogenization, the mixture was filtered through sterile three-layer gauze. After filtration, the prepared fecal microbiota suspension was mixed with basal medium at 1:9 (*v*/*v*) to obtain the final fecal fermentation broth. The basal medium was formulated according to a previous study [13], which contains the following components: peptone (2 g/L), yeast extract (2 g/L), NaHCO_3_ (2 g/L), MgSO_4_·7H_2_O (0.019 g/L), CaCl_2_·6H_2_O (0.019 g/L), KH_2_PO_4_ (0.049 g/L), K_2_HPO_4_ (0.049 g/L), L-cysteine (0.5 g/L), bile salts (0.5 g/L), NaCl (0.019 g/L), and Tween 80 (2 mg/L), 0.05 g/L of hemin and 10 μL/L of vitamin K. GP, GLP, and DOP were individually supplemented as carbon sources into 5 mL of fermentation broth at a final concentration of 10 mg/L. A substrate-free basal medium group was set as the negative control (NC). Each group was prepared in triplicate. All fermentation systems were incubated anaerobically at 37 °C for 24 h. Post-incubation samples were collected and stored at −80 °C for subsequent analyses.

### 2.8. Gut Microbiota Analysis

Total microbial genomic DNA was extracted from the fermentation samples using the Magnetic Soil and Stool DNA Kit (DP712, Tiangen Biotech, Beijing, China) according to manufacturer’s instructions. The purity and concentration of the extracted DNA were determined via a Qubit fluorometer (Invitrogen, Waltham, MA, USA), and DNA integrity was assessed by 1.0% agarose gel electrophoresis. The V3-V4 hypervariable regions of the bacterial 16S rRNA gene were amplified using the universal primer pairs 341F (5′-CCTACGGGNGGCWGCAG-3′) and 805R (5′-GACTACHVGGGTATCTAATCC-3′). The PCR protocol included an initial denaturation at 98 °C for 30 s, followed by 32 cycles of denaturation (98 °C, 10 s), annealing (54 °C, 30 s), and extension (72 °C, 45 s), with a final extension at 72 °C for 10 min. PCR products were purified with AMPure XP Beads (Beckman Coulter Genomics, Danvers, MA, USA) and quantified via Qubit (Invitrogen, USA). Qualified PCR products were evaluated using Illumina library quantification kits (Kapa Biosciences, Woburn, MA, USA) and an Agilent 2100 Bioanalyzer (Agilent, Santa Clara, CA, USA). Paired-end sequencing (2 × 250 bp) was performed on the NovaSeq 6000 platform using the NovaSeq 6000 SP Reagent Kit.

Sequencing primers were removed from de-multiplexed raw sequences using cut adapt (v1.9). Then, paired-end reads were merged using FLASH (v1.2.8). The low-quality reads (quality scores < 20), short reads (<100 bp), and reads containing more than 5% “N” records were trimmed by using the sliding-window algorithm method in fqtrim (v0.94). Quality filtering was performed to obtain high-quality clean tags according to fqtrim. Chimeric sequences were filtered using Vsearch software (v2.3.4). DADA2 was applied for denoising and generating amplicon sequence variants (ASVs). The sequence alignment of species annotation was performed by QIIME2 [14]. Plugin feature-classifier, and the alignment database was SILVA and NT-16S.

For downstream analyses, the ASV table was rarefied to 20,000 reads per sample to normalize sequencing depth. Alpha diversity (including Shannon, Chao1, and observed species indices) and beta diversity (Bray–Curtis dissimilarity, weighted and unweighted UniFrac) were calculated using QIIME 2.0. Differences in alpha diversity were assessed using the Kruskal–Wallis test followed by Dunn’s post hoc test with Benjamini–Hochberg false discovery rate (FDR) correction for multiple comparisons. Beta diversity dissimilarities were visualized via principal coordinate analysis (PCoA), and permutational multivariate analysis of variance (PERMANOVA, adonis function, 999 permutations) was used to test for significant differences between groups. LEfSe analysis was performed with an LDA threshold of 3.0 to identify differentially abundant taxa. Functional metagenome predictions were generated using Picrust2, and significantly different Kyoto Encyclopedia of Genes and Genomes (KEGG) orthologs were identified using the Wilcoxon rank-sum test with FDR correction.

### 2.9. Statistical Analysis

Data, including structural and fermentation indicators, were analyzed using GraphPad Prism 10 (GraphPad Software, San Diego, CA, USA). All results are presented as the mean ± standard deviation (SD) of three biological replicates. Prior to one-way analysis of variance (ANOVA), the assumptions of normality and homogeneity of variances were tested using the Shapiro–Wilk test and Levene’s test, respectively. Datasets that failed to satisfy ANOVA assumptions were subjected to log_10_ transformation. The ANOVA followed by Tukey’s post hoc multiple comparisons test was adopted for intergroup analyses, and a *p*-value < 0.05 was considered statistically significant.

## 3. Results and Discussion

### 3.1. Structural Properties of GP, GLP and DOP

#### 3.1.1. Mw Distribution and Monosaccharide Composition of GP, GLP and DOP

**Figure 1:**
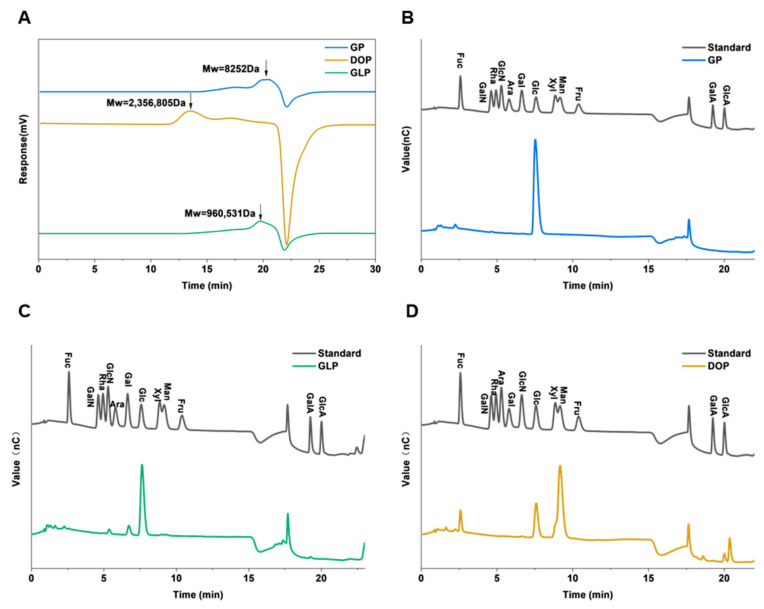
Molecular weight (Mw) and monosaccharide composition determination of GP, DOP and GLP. (**A**) HPSEC chromatograms of GP, DOP and GLP; (**B**–**D**) ion chromatograms of monosaccharide compositions in GP (**B**), GLP (**C**) and DOP (**D**).

The Mw and monosaccharide composition of GP, GLP, and DOP were shown in Figure 1 and Table 1. Regarding molecular weight distribution, GP exhibited an average molecular weight of 8252 Da, classifying it as a low-molecular-weight polysaccharide. In comparison, GLP and DOP had higher average Mw than GP, with average Mw of 960,531 Da and 2,356,805 Da, respectively (Figure 1A). These findings suggested the different molecular properties of GP, GLP, and DOP.

Monosaccharide composition analysis confirmed that GP, GLP, and DOP were heteropolysaccharides (Figure 1B–D and Table 1). Specifically, GP was predominantly composed of glucose at a molar ratio of 98.9%, with trace amounts of rhamnose (0.39%), glucosamine (0.30%), and arabinose (0.15%), which was consistent with a previous study [15]. Compared with GP, GLP exhibited a more complex monosaccharide profile, in which glucose remained the major constituent (83.24%), together with higher proportions of neutral sugars, including galactose (9.10%), arabinose (3.65%), and xylose (1.26%) [16]. In contrast, DOP showed a distinctly different composition characterized by a predominance of mannose (55.96%) and glucose (20.40%). It also contained considerable proportions of fucose (7.00%), glucuronic acid (8.71%), xylose (4.72%), galacturonic acid (2.80%), and a minor amount of galactose (0.33%), suggesting that it has the most complex monosaccharide composition of DOP among the three polysaccharides. The distinct monosaccharide profiles among these three polysaccharides may partly contribute to their divergent physicochemical properties, biodegradation behaviors, and bioactivities [17].

#### 3.1.2. FT-IR Analysis of GP, GLP and DOP

**Figure 2:**
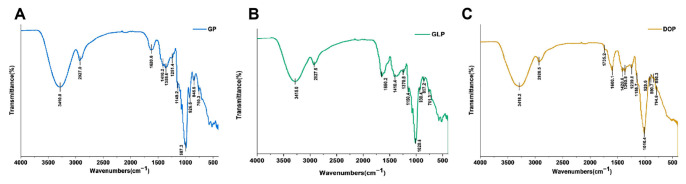
FT-IR spectra of GP (**A**), GLP (**B**) and DOP (**C**).

The FT-IR spectra of GP, GLP and DOP were shown in Figure 2. Overall, GP, GLP and DOP had the typical absorption peaks of polysaccharides [18]. A strong and broad absorption band centered at approximately 3420 cm^−1^ was observed in all spectra, corresponding to the stretching vibrations of O-H and N-H bonds in hydroxyl and amino groups [19]. The absorption peak at ~2920–2935 cm^−1^ was attributed to the C-H stretching vibrations, including methyl (-CH3), methylene (-CH2), and -CH groups. Absorption peaks in the range of 1600–1650 cm^−1^ corresponded to the C=O stretching or the bending vibration of adsorbed water [18]. The weak absorption peaks near 1410 cm^−1^ (GP and GLP) and 1420 cm^−1^ (DOP) represented an asymmetric C=O stretching vibration coupled with C-H bending, indicating the presence of uronic acids, consistent with the uronic acid content analysis [19].

Notably, distinct differences in peak positions and intensities were observed among the three polysaccharides. GP exhibited characteristic absorption bands at 926.5 cm^−1^ and 845.5 cm^−1^, indicating α-configuration glycosidic bonds (Figure 2A). For GLP, the characteristic peaks at 930.4, 857.2, and 761.3 cm^−1^ suggested the presence of α-glycosidic linkages (Figure 2B). In contrast, DOP displayed a characteristic absorption band near 1735 cm^−1^ assigned to O-acetyl carbonyl groups (Figure 2C), consistent with the structural features of acetylated glucomannan and its high mannose content [20]. Taken together, the FT-IR results revealed obvious structural differences in functional groups and sugar ring skeletons among the three polysaccharides, which may contribute to their divergent physicochemical and biological properties.

### 3.2. Characteristic of GP, GLP and DOP During Simulated Digestion

**Figure 3:**
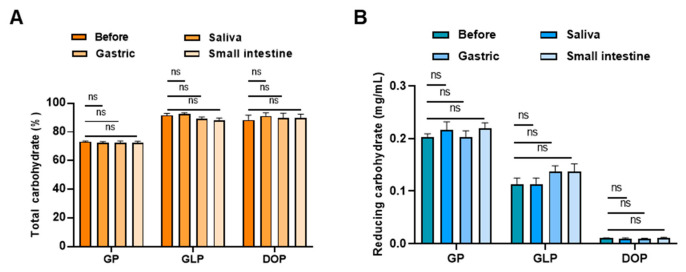
Changes in total carbohydrate and reducing carbohydrate contents of GP, GLP, and DOP during *in vitro* digestion. (**A**) Total carbohydrate levels of GP, GLP and DOP before digestion and after treatment with saliva, gastric juice, and small intestinal juice; (**B**) reducing sugar contents of GP, GLP and DOP before digestion and after treatment with saliva, gastric juice, and small intestinal juice. Data are presented as the mean ± standard deviation (SD) (*n* = 3); ns, not significant.

To investigate the gastrointestinal stability and *in vitro* digestibility of GP, GLP, and DOP during the human digestive tract, the three polysaccharides were subjected to sequential simulated digestion, including salivary, gastric, and small intestinal phases. As illustrated in Figure 3, there were no significant changes in the content of total carbohydrate or reducing carbohydrate levels. Consistently, their stability during *in vitro* digestion was reported in previous studies [21,22].

**Figure 4:**
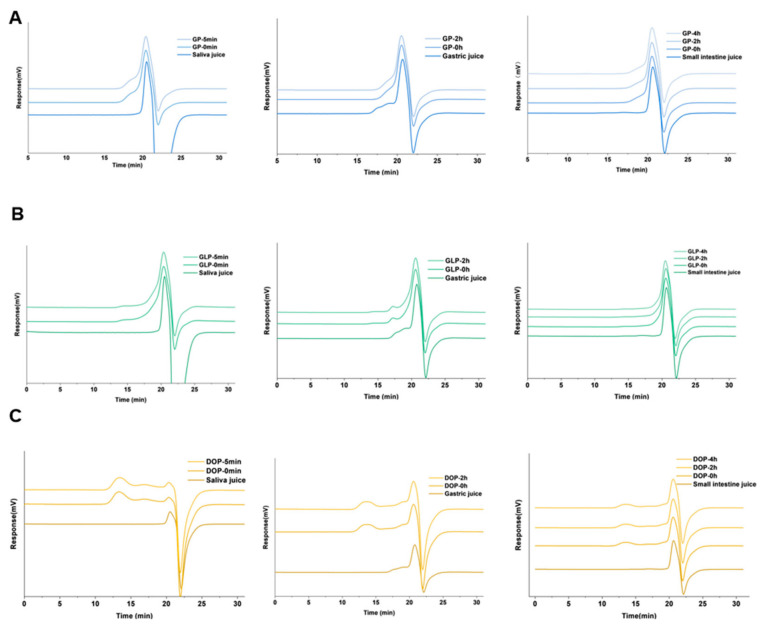
Molecular weight (Mw) changes in GP (**A**), GLP (**B**) and DOP (**C**) during *in vitro* digestion.

By further detecting Mw changes during salivary, gastric, and small intestinal digestion (Figure 4 and Table 2), we observed that the Mw of GP was significantly decreased after simulated digestion in the stomach and intestine, likely due to the partial acid hydrolysis of its glycosidic bonds in the gastric environment or the alkalinity during intestinal digestion [3]. In contrast, the GLP and DOP had no significant molecular weight changes, suggesting their remarkable structural stability throughout gastrointestinal digestion. This differential behavior can be explained by differences in their glycosidic linkage types, as β-type glycosidic linkages are highly resistant to digestion, whereas α-type glycosidic linkages can be effectively digested [23]. These findings indicate that GP is partially digested in the upper digestive tract, while GLP and DOP are highly resistant to digestive enzymes, and they can reach the large intestine for subsequent gut microbial fermentation.

### 3.3. Fermentation Properties of GP, GLP and DOP During In Vitro Fermentation

**Figure 5:**
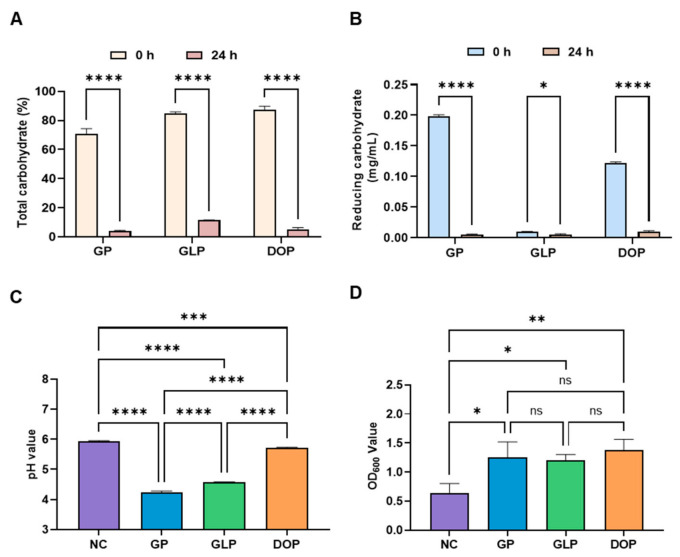
Fermentation characteristics of GP, GLP and DOP. (**A**) Total carbohydrate levels after *in vitro* fermentation for 24 h; (**B**) Reducing carbohydrate levels after *in vitro* fermentation for 24 h; (**C**) pH value; (**D**) OD_600_ value. NC, negative control; ns, not significant; * *p* < 0.05, ** *p* < 0.01, *** *p* < 0.001, **** *p* < 0.0001.

As shown in Figure 5A, the total carbohydrate contents of GP, GLP, and DOP decreased significantly after 24 h of *in vitro* fermentation, indicating that these polysaccharides were mainly degraded during fecal fermentation. Generally, gut microbiota release carbohydrate-active enzymes (CAZymes) to cleave the glycosidic bonds of polysaccharides and produce reducing sugars, which are further metabolized and utilized by the gut microbiota [24]. Accordingly, the reducing sugar levels in the GP, GLP, and DOP groups were significantly decreased at 24 h compared with the NC group.

During fermentation, gut microbiota consumes polysaccharides to proliferate and produce short-chain fatty acids (SCFAs), leading to changes in pH and OD600 value [25]. As such, alteration in pH value is an important index for evaluating the progression of fermentation [26], and the OD600 value reflected the proliferation of microorganisms [27]. As shown in Figure 5C, after 24 h of *in vitro* fermentation, the pH values of the GP, GLP, and DOP groups were significantly lower than the NC group (*p* < 0.0001), with the GP group showing the lowest pH, followed by GLP and DOP, indicating their different fermentation efficiency and SCFA production. These distinct pH values are primarily determined by their monosaccharide composition and glycosidic linkage patterns [17]. Furthermore, the elevated OD_600_ values in the polysaccharide groups demonstrated increased microbial biomass and the prebiotic potential of GP, GLP, and DOP. Collectively, GP, GLP, and DOP serve as favorable carbon sources for the gut microbiota, stimulating microbial growth and concomitant acid production during fermentation.

### 3.4. Different Regulation of GP, GLP and DOP on Gut Microbiota

**Figure 6:**
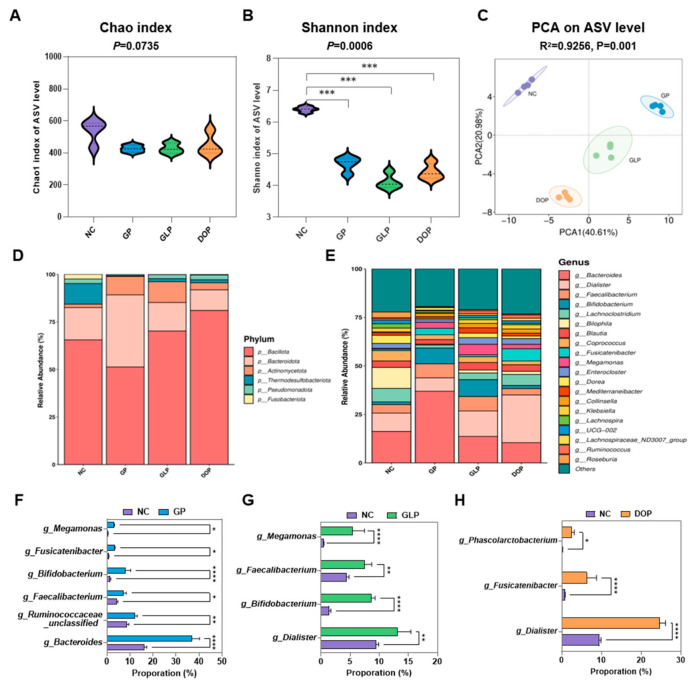
Regulation of GP, GLP and DOP on human intestinal microbiota. (**A**) Chao index; (**B**) Shannon index; (**C**) principal analysis (PCA); (**D**) microbial composition at the phylum level; (**E**) microbial composition at the genus level; (**F**–**H**) T-tests comparing highly abundant genus in GP, GLP and DOP separately.NC, negative control; * *p* < 0.05, ** *p* < 0.01, *** *p* < 0.001, **** *p* < 0.0001.

Gut microbial composition and functional diversity are key determinants of the metabolic capacity to utilize dietary polysaccharides within the dynamic intestinal ecosystem [28]. The regulatory effects of GP, GLP and DOP on gut microbiota were shown in Figure 6. Regarding the α-diversity, the indices reflecting species richness (Chao1 index) and evenness (Shannon index) were all decreased in the GP, GLP, and DOP groups (Figure 6A,B), indicating a notable reduction in gut microbial diversity upon polysaccharide intervention, and this may be related to the selective utilization of polysaccharides by specific gut microbiota [5,29]. The microbial composition change was further confirmed by the beta diversity analysis (ANOSIM *p* = 0.005, Figure 6C), which showed distinct clustering between the GP, GLP, DOP and NC groups, suggesting these polysaccharides induced profoundly different changes in gut microbial composition. Their distinctive molecular weight, glycosidic linkages, or monosaccharide composition might remodel the gut ecosystem toward a functionally specialized microbial profile [2,30].

By further microbiota composition analysis at the phylum level (Figure 6D), we found that all groups were dominated by *Bacillota* (formerly *Firmicutes*) and *Bacteroidota* (formerly *Bacteroidetes*), which is consistent with the well-characterized structure of the mammalian gut microbiota [31]. The genus-level structural characteristics in each group were analyzed in Figure 6E, and specifically microbial genera (relative abundance > 1%, *p* < 0.05) responsive to these polysaccharide interventions were identified in Figure 6F–H. Compared to the NC group, the GP group showed a comparatively high abundance of *Bacteroides*, *g_Ruminococcaceae_unclassified*, *Faecalibacterium* and *Bifidobacterium* (Figure 6F), consistent with known roles of these taxa in breaking down complex carbohydrates [32,33]. GLP treatment significantly elevated the abundance of genera such as *Dialister*, *Bifidobacterium*, *Faecalibaterium* and *Megamonas* (Figure 6G). Among these genera, *Megamonas* and *Dialister* are well-known polysaccharide degraders and SCFA producers [34,35], and co-enrichment of *Bifidobacterium* and *Faecalibacterium* suggests that GLP promotes cross-feeding between early colonizers and butyrogenic bacteria [36]. DOP uniquely enriched *Dialister*, *Fusicatenibacter*, and *Phascolarctobacterium* (Figure 6H), and *Phascolarctobacterium* has been reported to degrade polysaccharides [37,38]. Collectively, these findings highlight that structural differences among the three polysaccharides drive distinct gut microbial responses, providing a mechanistic basis for their differential health effects.

### 3.5. Functional Comparison of Gut Microbiota in GP, GLP and DOP

**Figure 7:**
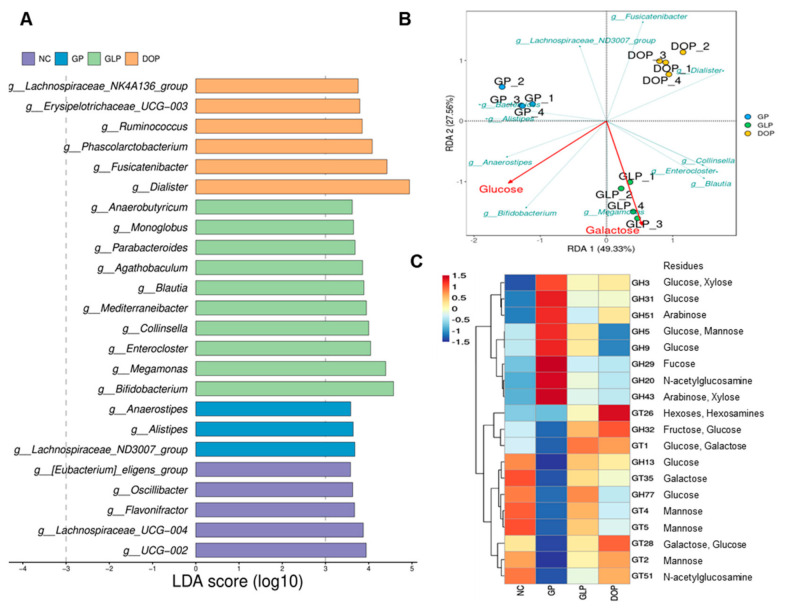
Unique microbial community structures, functional metabolic pathways, and CAZyme profiles in GP, GLP and DOP. (**A**) Indicator microorganisms with statistical difference at the genus levels by LEfSe analysis (LDA score > 3); (**B**) redundancy analysis (RDA) illustrating the correlation between gut microbiota and monosaccharide composition; (**C**) heatmap of PICRUSt-predicted carbohydrate-active enzyme (CAZyme) profiles.

To further investigate the specific regulatory effects of GP, GLP and DOP on gut microbiota composition, LDA effect size (LEfSe) analysis and statistically significant difference analysis were performed in Figure 7A. GP promoted the enrichment of *Anaerostipes*, *Alistipes*, and *Lachnospiraceae*. This enrichment is associated with the high glucose content of GP, which can serve as a readily fermentable substrate for *Lachnospiraceae* [39], while *Anaerostipes* thrived via cross-feeding with lactate- and acetate-producing genera during glucose fermentation. For GLP treatment, a diverse array of genera, such as *Bifidobacterium*, *Megamonas*, and *Blautia*, were specifically enriched. *Bifidobacterium* spp. Preferentially utilizing glucose, galactose, and L-arabinose, GLP, rich in arabinose and galactose side chains, acted as metabolic signals that induce specific glycoside hydrolases in *Bifidobacterium* [40] and subsequently cross-feed butyrate producers such as *Blautia* and *Megamonas* [41]. In contrast, DOP induced a highly specific microbial enrichment pattern dominated by *Dialister* accompanied by *Fusicatenibacter*, *Phascolarctobacterium*, *Ruminococcus*, and *Lachnospiraceae_NK4A136.* These enriched genera are capable of directly utilizing mannose and uronic acid or in the manner of cross-feeding [42,43], which explains their selective enrichment by DOP due to its unique mannose- and uronic acid-rich monosaccharide composition.

To decipher possible reasons for the distinctive genus of GP, GLP and DOP, we conducted redundancy analysis (RDA) to investigate the correlations between their signature monosaccharides (glucose, galactose, arabinose, mannose, and galacturonic acid) and significantly enriched genera. As shown in Figure 7B, glucose and galactose were positively correlated with significant genera. Notably, the positive correlation between the GP group and glucose demonstrated that GP’s high glucose content selectively promotes the growth of *Bacteroides*, *Bifidobacterium*, and *Alistipes* species. The GLP group was associated with galactose and induced an alternative metabolic niche favoring taxa possessing specialized galactose metabolism pathways, such as *Blautia*, *Collinsella*, and *Megamonas*. Conversely, the DOP group exhibited limited correlation with either glucose or galactose and clustered with *Fusicatenibacter* and *Lachnospiraceae* members, implying that the high mannose content of DOP may distinguish it from GP and GLP. Collectively, these findings demonstrate that monosaccharide composition is a critical determinant of the prebiotic efficacy of polysaccharides.

The PICRUSt2 tool was used to predict the functional potential of microbial communities, specifically the relative abundance of CAZyme gene families (top 20) in the GP, GLP, and DOP groups. As shown in Figure 7C, the GP group exhibited a predicted enrichment of glycoside hydrolase (GH) families targeting glucose-containing substrates, including GH3, GH31, GH5, GH9, GH20, GH29, and GH43. The inferred CAZyme profile of the GLP group shows moderate upregulation of glucose-targeting GH families (GH3, GH31, GH5, GH9) together with galactose-associated glycosyltransferase (GT) families (GT1, GT35, GT28), which is consistent with its high glucose content and characteristic galactose and arabinose components. Notably, the DOP group displayed a unique CAZyme signature, with significant enrichment of GT families targeting mannose and hexosamine metabolism (GT26, GT2, GT51, GT4, GT5) alongside elevated fructose/glucose-specific GH32. Such differential predicted CAZyme patterns were correlated with the high contents of mannose and glucose in DOP. Taken together, these findings suggest that monosaccharide composition influences CAZyme signatures, which may further mediate gut microbial community structure and prebiotic efficacy.

## 4. Conclusions

In the present study, we demonstrated that GP, GLP and DOP are resistant to upper gastrointestinal digestion, exhibit distinct fermentation characteristics, and exhibit divergent modulatory effects on the gut microbial community. More importantly, our findings highlight that monosaccharide composition acts as a crucial structural determinant of polysaccharides, governing their fermentation efficiency and prebiotic specificity, thus providing a mechanistic basis for their precision application as prebiotic ingredients. However, several limitations exist in our work. Firstly, pooled fecal samples from multiple volunteers were used in the *in vitro* fecal fermentation to eliminate inter-individual microbial variation, which reflects a population-averaged response to polysaccharide intervention. Future studies using individual fecal samples to perform polysaccharide fermentation separately are needed to further explore inter-individual microbial responsiveness and heterogeneity. Secondly, the above conclusions are mainly based on static *in vitro* simulated digestion and fermentation models, which cannot fully replicate the dynamic physiological environment of the human gastrointestinal tract, such as intestinal peristalsis, real pH fluctuation, mucosal barrier and complex host–microbe interactions, and future studies are needed to assess their digestion and microbial metabolism *in vivo*. Thirdly, the specific microbial metabolic pathways responsible for the degradation, utilization and differential regulation of three polysaccharides remain unclarified, and multi-omics strategies such as metagenomics and transcriptomics are still required to further elaborate the underlying microbial metabolic mechanisms. Despite these limitations, our findings provide new insights into the structure-fermentation relationship and potential application of GP, GLP, and DOP in shaping gut microbial homeostasis.

## Figures and Tables

**Table 1:** Monosaccharide composition of GLP, DOP and GP (molar ratio, %).

	GP	GLP	DOP
Fucose	ND	0.41 ± 0.009	7.00 ± 0.12
Galactosamine	ND	ND	ND
Rhamnose	0.39 ± 0.006	0.48 ± 0.007	ND
Arabinose	0.15 ± 0.003	3.65 ± 0.06	ND
Glucosamine	0.30 ± 0.005	ND	ND
Galactose	ND	9.10 ± 0.05	0.33 ± 0.005
Glucose	98.90 ± 1.81	83.24 ± 1.36	20.40 ± 0.48
Xylose	ND	1.26 ± 0.008	4.72 ± 0.07
Mannose	ND	1.07 ± 0.01	55.96 ± 1.13
Fructose	ND	ND	ND
Galacturonic acid	ND	0.78 ± 0.01	2.80 ± 0.04
Glucuronic acid	ND	ND	8.71 ± 0.16

ND: not detected; data are presented as the mean ± standard deviation (SD) (*n* = 3).

**Table 2:** Molecular weight (Mw) changes in GP, GLP and DOP during *in vitro* simulated digestion.

	GP (×10^3^ Da)	GLP (×10^6^ Da)	DOP (×10^6^ Da)
Saliva			
0 min	8.15 ± 0.44 ^a^	0.96 ± 0.19 ^a^	2.33 ± 0.31 ^a^
5 min	8.06 ± 0.37 ^a^	0.93 ± 0.26 ^a^	2.31 ± 0.35 ^a^
Gastric			
1 h	6.47 ± 0.51 ^b^	0.88 ± 0.22 ^a^	2.16 ± 0.28 ^a^
Small intestine			
2 h	5.88 ± 0.46 ^c^	0.85 ± 0.33 ^a^	2.07 ± 0.31 ^a^
4 h	5.66 ± 0.39 ^c^	0.81 ± 0.18 ^a^	2.05 ± 0.40 ^a^

Data are presented as the mean ± standard deviation (SD) (*n* = 3); different lowercase letters within the same column indicate significant differences (*p* < 0.05) across different digestion stages.

## Data Availability

The original contributions presented in this study are included in the article. Further inquiries can be directed to the corresponding author.

## Abbreviations

The following abbreviations are used in this manuscript

GP	*Ginseng* polysaccharide
GLP	*Ganoderma lucidum* polysaccharide
DOP	*Dendrobium officinale* polysaccharide
Mw	Molecular weight
CAZyme	Carbohydrate-active enzyme
GH	Glycoside hydrolase
GT	Glycosyltransferase
RDA	Redundancy analysis
